# 40 years neonatology: An academic life

**DOI:** 10.1007/s00508-024-02360-2

**Published:** 2024-04-18

**Authors:** Arnold Pollak

**Affiliations:** Department of Pediatrics and Adolescent Medicine, Division of Neonatology, Pediatric Intensive Care Medicine and Neuropediatrics, Medical University of Vienna, Vienna, Austria

**Keywords:** Neonatology, Carbohydrate metabolism, Neonatal anemia, Toxoplasmosis screening program, Neonatal screening program

## Abstract

**Background:**

A complete review of the development of neonatology in the last 40 years would probably require a compendium with several volumes, to bring to view the remarkable improvements in survival rates and neurodevelopmental outcomes of ill babies in Austria, most industrial countries and to some extent worldwide. The challenge I had to solve here was to integrate my own contributions to the field of neonatology during this period and particularly the contributions of my team from the Division of Neonatology and Pediatric Intensive Care Medicine, Department of Pediatrics and Adolescence Medicine, Medical University Vienna where I was working first as an intern and resident and later had the privilege to become head of department.

**Aim:**

This very personal review was conceived to showcase the milestones of neonatology where, in my opinion, our department made some meaningful contributions in research and clinical practice during the past 40 years.

**Methods:**

A total of 10 areas of interest were selected which most likely influenced survival rates of preterm infants born at increasingly younger gestational ages and ameliorated long-term clinical and neurodevelopmental outcomes, including:

1) Construction and continuous modernization of neonatal intensive care units (NICUs). 2) Installation of the “Regionalization Program for NICUs in Vienna”. 3) Treatment of respiratory distress syndrome (RDS) of premature babies. 4) Fine tuning of glucose metabolism for growth and outcome. 5) Neurodevelopmental care. 6) Neonatal hematology. 7) Infection control. 8) The toxoplasma screening program. 9) The newborn screening program. 10) Quality control: the Vermont Oxford Neonatal Network (VONN).

**Results:**

Over the past four decades advancements in research and technology have allowed a transformative development of neonatal medicine. Survival rates without increased morbidity for very premature infants with gestational ages reaching to what we consider nowadays the border of viability have constantly increased. In my professional life as a neonatologist in Austria I have had the possibility to support and shape some of these developments together with my team.

**Conclusion:**

As we look ahead it is imperative to build upon the progress made, harnessing the power of science and technology to further improve the survival and quality of life for preterm infants in Austria and worldwide. At the same time, neonatology must continue to prioritize ethical reflection and education, fostering a culture of integrity, interdisciplinary collaboration, and the development of guidelines and protocols that uphold ethical standards while addressing the evolving needs and complexities of neonatal medicine.

## Introduction

Over the last decades neonatology and pediatric intensive care medicine made significant advancements, probably more than most other medical specialties, which have substantially improved the survival rates of preterm infants born at increasingly younger developmental ages and enhanced long-term clinical outcomes.

It is difficult to objectively rank these achievements in terms of biomedical importance and their significance for population health. Having had the honor of heading a thriving academic Division of Neonatology for more than 22 years (1 May 1992–1 October 2014) enables me to shine the light on a personal selection of notable accomplishments of medical care and research in the field of modern neonatology, contributed in part by this Division of Neonatology at the Department of Pediatrics of the Medical University Vienna, Austria.

**Fig. 1:**
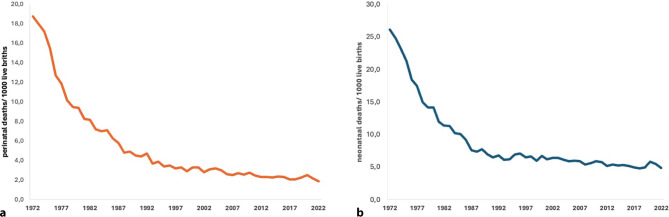
Development of perinatal and neonatal mortality in the past four decades. **a** Perinatal, **b** neonatal deaths per 1000 live births in Austria from 1972 to 2022 (data derived from Statistics Austria)

Before presenting these achievements, enabled by substantial progress in research and technology, against the background of the steady decrease of perinatal and neonatal mortality in Austria over the past decades (Fig. 1), I would like to commence by quoting David M. Cutler [1]: “The technology of birth: is it worth it?”

“We evaluate the costs and benefits of increased medical spending for low-birthweight infants. Lifetime spending on low-birthweight babies increased by roughly $ 40,000 per birth between 1950 and 1990. The health improvements resulting from this have been substantial. Infant mortality rates fell by 72% over this time period, largely due to improved care for premature births. Considering both length and quality of life, we estimate the rate of return for care of low-birthweight infants at over 500%. Although prenatal care and influenza shots are more cost-effective than neonatal care, it is significantly more cost-effective than other recent innovations, such as coronary artery bypass surgery, treatment of severe hypertension, or routine Pap smears for women aged 20 to 74. We conclude that the answer to the question posed in this paper is a resounding yes.” [1].

## Neonatal intensive care units

The development of neonatal intensive care units (NICUs) equipped with advanced monitoring and therapeutic technologies had an enormous impact on survival rates and outcomes of critically ill newborns. The NICUs facilitate early detection and intervention for various health complications, ensuring timely treatment and reducing the risk of long-term negative outcomes particularly of premature infants but also of term newborns with congenital or acquired illnesses or malformations The establishment and continuous improvement of NICUs rests on a number of substantial cornerstones of technological advancements, which importantly include the discovery of “continuous positive airway pressure (CPAP)” as non-invasive method for treating respiratory distress syndrome (RDS) and preventing the need for mechanical ventilation. This concept, which was first introduced by George Gregory, a pioneer of modern neonatology in 1971 [2], revolutionized the treatment of premature infants with respiratory failure.

Further continuous improvements in technology and modes of mechanical ventilatory support have made intensive care treatment safer and more effective. Of great importance in this context was the recognition of oxygen as a potential harmful drug for brain, eyes, lungs and other tissues during treatment when hypoxic or hyperoxic insults were not prevented [3–10].

## Introduction of perinatal centers

In a next step for improving clinical outcomes after premature birth and neonatal morbidities I had the opportunity to initiate the regionalization concept of neonatal intensive care for Vienna. This unique approach included the categorization of NICUs into different levels (level I–level IV) based on the intensity of care they can provide, with level IV NICUs being constructed and equipped for treating the most complex cases, including surgical interventions.

A major goal of the concept was to transport women at risk for developing ill neonates prior to delivery to specialized perinatal care centers instead of transporting newborns with life-threatening conditions after birth. This development has been crucial in further enhancing the options for providing timely and appropriate care for severely sick and high-risk infants.

## Respiratory distress syndrome (RDS)

### Surfactant therapy: a milestone of neonatal medicine

The introduction of surfactant therapy for premature infants with RDS significantly improved outcomes by reducing the severity of the disease and its complications. Surfactant therapy, which involves administering exogenous surfactant directly into the lungs of affected newborns to restore proper lung function and improve gas exchange, significantly alleviates respiratory distress, enhances oxygenation, and reduces the need for mechanical ventilation, thereby lowering the risk of associated complications and improving the overall prognosis for infants affected by RDS. The development of different surfactant preparations and the understanding of optimal dosage and timing have improved its effectiveness even further in preventing and treating RDS [11, 12].

### Extracorporeal membrane oxygenation

Extracorporeal membrane oxygenation (ECMO) was developed as an artificial replacement of pulmonary function in cases of severe RDS with respiratory failure; however, as conventional treatment for severe RDS advanced, the need for pulmonary ECMO, an invasive and expensive treatment modality, diminished. The ECMO was then more required for circulatory support by means of the AREC (“assistence respiratoire extra-corporelle”), a veno-venous system, prior to and after complex cardiosurgical interventions. We showed that the use of AREC results in rapid improvement of oxygenation in children with congenital heart defects when high pulmonary arterial resistance in cyanotic heart defects prevents lung perfusion. Up to 80% of ECMOs performed in our pediatric intensive care unit (PICU), located next to the NICU, were for circulatory failure. Other indications were congenital diaphragmatic hernia, severe sepsis with organ failure, combined circulatory and respiratory failure after resuscitation etc.

Our research was directed towards improving ECMO techniques such as using nonocclusive pumps, portable devices, small priming volumes and tapered anticoagulation protocols to enable survival through ECMO even under virtually hopeless hemodynamic conditions.

Much effort was made on cerebral functioning monitoring to prevent permanent neurological impairment resulting from severe pre-ECMO hypoxia [13–18].

### Prevention of severe RDS—Antenatal steroid therapy

While the availability of surfactant therapy constituted a milestone in the treatment of RDS, parallel developments in the prevention constituted a major progress towards better outcomes of prematurely born infants. As such, antenatal steroid therapy stands as a cornerstone in the management of women at risk for preterm delivery, playing a crucial role in improving lung maturation and reducing the incidence and severity of RDS in premature infants. Administered to pregnant women at risk of preterm birth between 24 and 34 weeks of gestation, antenatal steroids accelerate fetal lung maturation by promoting the production of surfactant and enhancing the structural development of the lungs. This therapy has been extensively studied and proven to significantly decrease the risk of RDS and its complications as well as overall neonatal mortality [19]. The beneficial effects of antenatal steroid therapy further extend beyond respiratory outcomes, contributing to improved neurodevelopmental outcomes in preterm infants thus constituting a vital component of prenatal care for women at risk of preterm delivery with profound impact on neonatal health and long-term outcomes.

## Carbohydrate metabolism

### Fine tuning glucose regulation

Fine tuning of glucose regulation is of paramount importance for premature and low birth weight infants (LBWI) and constituted a major focus of our scientific and clinical work and contributions. Balanced parenteral alimentation is a vital component of the nutritional management for LBWI, particularly those born prematurely who may have initially immature digestive systems or inadequate oral intake. By this approach essential energy and nutrients are provided to meet metabolic demands and support growth and development of these infants. Glucose serves as the primary fuel source for the brain and other vital organs, playing a critical role in maintaining normal physiological functions and promoting weight gain. As such careful monitoring of blood glucose levels is essential to prevent impaired glucose homeostasis resulting in hypoglycemia followed by gluconeuropenia or hyperglycemia associated with glucosuria, osmotic diuresis. which are equally important risk factors for neurodevelopmental impairment. Our group contributed to this important topic with several clinical as well as experimental studies.

We studied glucose disposal of LBWIs under steady-state hyperglycemia; the effect of exogenous insulin on glucose disposal in LBWIs, renal functions of LBWI with hyperglycemia, sympatho-adrenal response to hypoglycemia, hypoglycemia and congenital growth hormone deficiency, cord blood insulin levels, morbidities in infants of diabetic mothers, studies on hyperinsulinemia/nesidioblastosis, neonatal islet cell adenoma and gluconeogenesis in experimental intrauterine growth retardation in rats [20–29].

### Glycosylated hemoglobin (Hba1c)

In the late 1970s we were among the first groups worldwide to develop tools for evaluating glycohemoglobin as a parameter for an integrated glucose control measurement superior to spot blood or urinary glucose measurement. We first established a simple colorimetric assay (TBA [Thiobarbituric Acid assay]) for monitoring total glycohemoglobin and later set up a liquid chromatography method for measuring glycosylated hemoglobin (Hba1c). Nowadays, HbA1c is accepted as THE classical routine marker for evaluating metabolic control and guiding therapy in diabetic patients.

Our primary focus was glucose control in diabetic pregnancies, the main relevant factor for an overall improvement of pregnancy outcome for these high-risk infants.

In addition, we studied HbA1c in children and juveniles with type l diabetes and nonenzymatic glycosylation of various proteins, peptides and tissues such as umbilical cord collagen and glomerular basement membranes [30–45].

## Neurodevelopmental care

Increased awareness of the importance of neurodevelopmental care, including strategies to support brain development and minimize stress in the NICU environment, has led to improved long-term outcomes and quality of life for premature infants. This specialized care involves a combination of evidence-based strategies tailored to the unique needs of each infant, with a primary focus on promoting a nurturing and developmentally supportive environment. Key components include minimizing exposure to excessive light and noise, providing gentle handling and skin-to-skin contact through kangaroo care, promoting breastfeeding or appropriate nutritional support, and implementing developmental positioning to support motor development and reduce the risk of musculoskeletal issues [46–56].

Further developments have led to the inclusion of comprehensive follow-up visits, ensuring continued support for infants’ optimal brain and behavioral development. In 1994 we introduced follow-up visits in our institution, involving multidisciplinary teams comprising neonatologists, developmental specialists, physical therapists and other healthcare professionals to monitor infants’ growth and neurodevelopmental progress through standardized developmental screening and assessments tools. As such, these follow-up visits serve as critical opportunities to monitor infants’ progress, to promptly address developmental delays or concerns and tailor interventions and support services accordingly. Maintaining a continuum of care from the NICU to outpatient settings allows improvement of long-term outcomes and promoting the most beneficial development of preterm and high-risk infants beyond the neonatal period.

## Hematology

### Neonatal anemia

Critically ill premature infants frequently receive blood transfusions for anemia as a result of laboratory phlebotomies during intensive care treatment. In these infants the endogenous red blood cell production (RBC) cannot cope with the occurred blood losses. Prematurely born infants are particularly susceptible to the consequences of neonatal anemia including impaired oxygen delivery to tissues, decreased growth and development, and an increased risk of morbidity and mortality. Clinical trials of erythropoietin (EPO) treatment in this patient group have shown only moderate success in reducing RBC transfusions, which led our group to postulate the hypothesis that depleted iron stores or limited bioavailability of iron for hemoglobin synthesis might be the underlying reason and we developed several clinical trials to address this question. We were able to show that in LBWI a combined therapy of EPO, Fe, folate, and vitamin B12 during the first weeks reduces the need for RBC transfusion [57–63].

### Fetal hemolytic disease

Fetuses with erythrocyte alloimmune disease are at risk for developing life-threatening anemia. Because carbon monoxide (CO) is a byproduct of heme degradation we studied the concentration of CO in fetal blood as HbCO. We showed that increased HbCO levels detected in fetuses with erythrocyte alloimmunization are the result of accelerated hemolysis.

Further studies are necessary to decide if HbCO in fetal blood could be used for guiding therapeutic interventions in pregnancies affected by alloimmune disease.

We assessed whether whole blood HbCO and plasma bilirubin, two indicators of hemolysis, are elevated in infants with severe Rh− isoimmune hemolytic disease during the first months of life. Our study showed that among these infants, elevated total bilirubin levels and COHb/Hb ratios identified in the early weeks of life indicate continuing hemolysis due to persistence of maternal Rh antibodies. In another study, we showed that differences in the antepartum fetomaternal HbCO relationships in control and alloimmunized groups reflect increased endogenous CO production among alloimmunized fetuses as a result of pathologic hemolysis [64–66].

## Infection control

Prenatal, i.e., intrauterine and early as well as late onset postnatal infections and septicemia, remain a major cause of mortality and morbidity rates among premature infants despite the use of modern anti-infective therapy. Especially extremely preterm infants and ill infants requiring intensive care invasive treatment are prone to systemic infections compromising multiple organs, due to their immature immune defense system. Infections can occur through various routes, including vertical transmission from the mother, hospital-acquired infections or community exposure. The consequences of infection in premature infants can be severe, ranging from systemic inflammatory response syndrome (SIRS) to sepsis, meningitis, pneumonia, and necrotizing enterocolitis (NEC). These conditions not only pose immediate threats to life but also increase the risk of long-term neurodevelopmental impairments, such as cerebral palsy, cognitive deficits, and sensory impairments.

Consequently, infection control was the major target of our interventions since the early 1970s until today when neonatal care was rapidly advancing and premature and LBWI of decreasing gestational ages, nowadays down to 22/23 weeks, survived without or with minor neurological sequelae. Members of our department were involved in a large number of clinical, laboratory, molecular and immunological studies in this area [63, 67–82].

## Toxoplasmosis screening program: a success story introduced by Otto Thalhammer

Although gestational infection with the parasite *Toxoplasma gondii* is a significant health problem worldwide, there is no standardized diagnostic and therapeutic approach.

Fetal infection and congenital toxoplasmosis are the result of maternal-fetal transmission among previously seronegative women who acquire acute infection during pregnancy requiring treatment until delivery. Infected infants are often asymptomatic at birth (= congenital *Toxoplasma* infection) or may manifest as a wide range of signs and symptoms, including intracerebral calcification, hydrocephalus, retinochoroiditis resulting in severe visual and cognitive impairment (= congenital toxoplasmosis).

Austria and France were the first European countries to introduce, and are still, the only countries with a nationwide prenatal serological toxoplasmosis screening program. In Austria it began in 1974 in response to the high incidence of 7.8 infected infants per 1000 live births [83, 84]. In 1992 we introduced an Austrian Register for Toxoplasma Infection in pregnancy for determining the actual incidence of primary gestational infections with *Toxoplasma gondii* and congenital toxoplasmosis. Annually, 8.5 per 10,000 women acquired *Toxoplasma* infection during pregnancy, and 1.0 per 10,000 infants had congenital *Toxoplasma* infection (13% mean transmission rate) We showed that women treated according to the Austrian scheme had a sixfold, decrease in the maternal-fetal transmission rate compared to untreated women.

We also showed that the screening program is economically justified.

We introduced for all newborns of infected mothers a routine follow-up program until age 12 months including 3‑monthly serological tests using the Sabin-Feldmann dye (still the gold standard test), brain ultrasound scans, ophthalmologic examinations, psychological testing of cognitive and other developmental skills.

Moreover, we were the second group worldwide, who introduced PCR testing for the *Toxoplasma* agent in amniotic fluid as the specific and direct test for confirming or excluding fetal infection when primary gestational infection had been detected within the screening program. This was important because it determined the choice of maternal medication.

If the parasite had already reached the fetal compartment, only medications which are able to cross the placenta barrier can be effective [85–105].

## The newborn screening program for Austria

This nationwide program for congenital metabolic and endocrinologic disorders was introduced in 1966 by Otto Thalhammer as the so-called Vienna test program for the early detection of phenylketonuria [106]. It constitutes a critical public health initiative aimed at identifying infants at risk of certain inherited conditions early in life, allowing for timely intervention and treatment. This program typically involves the collection of blood samples from newborns shortly after birth, which are then analyzed for various metabolic and endocrine disorders. Over time, the Austrian initiative evolved by expanding the screening panel from phenylketonuria (PKU) and galactosemia to congenital hypothyroidism, biotinidase deficiency, cystic fibrosis, and congenital adrenal hyperplasia. In 2002, the introduction of tandem mass spectrometry (MS/MS) substantially increased the number of detectable inborn errors of metabolism and now includes disorders of fatty acid oxidation, organic acidurias and various disorders of amino acid metabolism and recently (one of the first in Europe): spinal muscular atrophy (SMA) and severe congenital immune deficiencies (SCID). Early detection through newborn screening enables the timely recommendation of appropriate interventions, such as dietary modifications, hormone replacement therapy, gene therapy or other medical interventions, before symptoms manifest, thus preventing serious complications and mitigating the long-term impact of these conditions on the infant’s health and development [107–109].

## Quality control: the Vermont Oxford Neonatal Network (VONN)

The VONN is an internet-based quality improvement collaborative.

Its mission is: “To improve the quality, safety and value of care for newborn infants and their families through a coordinated program of data-driven quality improvement, education and research” (https://public.vtoxford.org/).

We realized early that treatment according to international quality standards and continuous quality improvement is of upmost importance for providing neonatal medicine in its highest standard. Internal quality control through review of hospital statistics is limited because of the small number of patients per center. Thus, evaluation of different clinical management strategies is nearly impossible unless individual center results are continuously assessed and monitored in a standardized way and compared with international standards. For this reason, our center for high-risk neonates decided to participate in the VONN, the biggest and most comprehensive neonatal database worldwide. In 1994 we were the first European (and non-US) center to become a member of the VONN [5]. Meanwhile, 1400 centers worldwide have joined. The reporting tools give centers the necessary data and benchmarks to make meaningful improvements. Since 1994 we continue each year to participate and contribute to this excellent and important quality control system. Our example was later followed by almost all national and European international centers treating high-risk neonates mainly VLBWI.

## Conclusion

Over the past four decades, advancements in research and technology have allowed a transformative development of neonatal medicine. Survival rates without increased morbidity for very premature infants with gestational ages reaching to what we consider nowadays the border of viability have constantly increased.

Prenatal care has evolved to include routine screening, risk assessments and interventions aimed at identifying and managing conditions that predispose mothers to preterm labor or fetal complications. The Austrian social and healthcare system has allowed improvements in the national organization and administration of prenatal, perinatal and postnatal care systems to reduce risk factors associated with neonatal mortality and improve long-term outcomes of affected infants.

In my professional life as a neonatologist in Austria I have had the possibility to support and to shape some of these developments in my position as Head of the Division of Neonatology at the Medical University of Vienna.

Current state-of-the-art medical technologies and staffed by skilled healthcare professionals, now provides round-the-clock monitoring, specialized therapies, and developmental support. Moreover, past ongoing research endeavors with our contributions have deepened our understanding of the intricate mechanisms underlying the most severe and complex neonatal morbidities, such as those associated with metabolic complications, respiratory and hematologic pathologies and their comorbidities and associated adverse consequences, which have and are continuing to pave the way for new targeted interventions and preventive strategies.

Despite these remarkable advancements, challenges persist, including the persistent rates of prematurity, socioeconomic disparities in neonatal outcomes, and the long-term consequences of preterm birth on neurodevelopment and overall population health. Equally important and challenging for clinicians and nursing staff are the various ethical and cultural issues always apparent in neonatal intensive care units. In my view and as stated by others [110], senior and junior staff should concede for themselves more space for reflecting and perhaps changing ethical attitudes including communication strategies with parents.

As we look ahead, it is imperative to build upon the progress made, harnessing the power of science and technology to further improve the survival and quality of life for preterm infants in Austria and worldwide. At the same time, neonatology must continue to prioritize ethical reflection and education, fostering a culture of integrity, interdisciplinary collaboration, and the development of guidelines and protocols that uphold ethical standards while addressing the evolving needs and complexities of neonatal medicine.
